# Functional Characterization of a Gene in *Sedum alfredii* Hance Resembling *Rubber Elongation Factor* Endowed with Functions Associated with Cadmium Tolerance

**DOI:** 10.3389/fpls.2016.00965

**Published:** 2016-06-29

**Authors:** Mingying Liu, Wenming Qiu, Xuelian He, Liu Zheng, Xixi Song, Xiaojiao Han, Jing Jiang, Guirong Qiao, Jian Sang, Mingqing Liu, Renying Zhuo

**Affiliations:** ^1^State Key Laboratory of Tree Genetics and Breeding, Chinese Academy of Forestry, BeijingChina; ^2^Key Laboratory of Tree Breeding of Zhejiang Province, The Research Institute of Subtropical Forestry, Chinese Academy of Forestry, HangzhouChina; ^3^Biotechnology Research Center of China Three Gorges University, YichangChina; ^4^Vocational Secondary Specialized School of Hedong District, LinyiChina

**Keywords:** cadmium, Cd tolerance, hyperaccumulator, *rubber elongation factor* (*REF*), *Sedum alfredii* Hance

## Abstract

Cadmium is a major toxic heavy-metal pollutant considering their bioaccumulation potential and persistence in the environment. The hyperaccumulating ecotype of *Sedum alfredii* Hance is a Zn/Cd co-hyperaccumulator inhabiting in a region of China with soils rich in Pb/Zn. Investigations into the underlying molecular regulatory mechanisms of Cd tolerance are of substantial interest. Here, library screening for genes related to cadmium tolerance identified a gene resembling the *rubber elongation factor* gene designated as *SaREFl*. The heterologous expression of *SaREFl* rescued the growth of a transformed Cd-sensitive strain (*ycf1*). Furthermore, *SaREFl*-expressing *Arabidopsis* plants were more tolerant to cadmium stress compared with wild type by measuring parameters of root length, fresh weight and physiological indexes. When under four different heavy metal treatments, we found that *SaREFl* responded most strongly to Cd and the root was the plant organ most sensitive to this heavy metal. Yeast two-hybrid screening of *SaREFl* as a bait led to the identification of five possible interacting targets in *Sedum alfredii* Hance. Among them, a gene annotated as *prenylated Rab acceptor 1* (*PRA1*) *domain protein* was detected with a high frequency. Moreover, subcellular localization of SaREF1-GFP fusion protein revealed some patchy spots in cytosol suggesting potential association with organelles for its cellular functions. Our findings would further enrich the connotation of *REF-like* genes and provide theoretical assistance for the application in breeding heavy metal-tolerant plants.

## Introduction

Nowadays, as we are enjoying the conveniences brought by industrial developments, we are also being challenged with the threats of environmental contaminations caused by hazardous environmental pollutants (Shahid et al., 2014). Among these pollutants, the heavy metals, a loosely defined group of elements exhibiting metallic properties (Rascio and Navari-Izzo, 2011), are exerting persistent potential harmful effects on the environment and living beings as they might be bioaccumulated in food webs because of difficulties in detoxification and long residence times in soils (Ahmad and Ashraf, 2011). Those elements that resemble essential minerals, such as mercury (Hg), lead (Pb), and cadmium (Cd), are deemed to be more threatening as they are more probable to enter the cell through the existing mineral uptake machinery (Lin and Aarts, 2012). As a Class B metal, cadmium represents a major toxic heavy metal and cadmium pollution has aroused worldwide concerns because excessive Cd exposure would cause emphysema and osteoporosis, leading to irreversible damages to lungs, kidneys, and bones in humans (Straif et al., 2009). When plants encounter with an excess level of a heavy metal, they will display several biological and physiological changes including reduced biomass, leaf chlorosis, inhibited root growth, morphological alterations, and more seriously plant death (Yadav, 2010; Su et al., 2014).

Based on the previous studies, the mechanisms utilized by plants to resist the toxic effect of a heavy metal can be summarized into two major strategies (Verbruggen et al., 2009a; Anjum et al., 2012). The first one is the excluder strategy, in which the plants attempt to prevent heavy metals from entering the roots, for instance by limiting metal bioavailability or by regulating expression of proteins involved in assimilating and transporting metals (Lin and Aarts, 2012). The other strategy is the tolerance tactics, which rely on the coordination and maintenance of homeostasis, for instance by controlling of metal ions through compartmentalization and detoxification and elevating expression of stress-related genes (Zhang et al., 2001; Lin and Aarts, 2012). These ecophysiological adaptations to metalliferous environment produce heavy-metal hypertolerant plants which can withstand exposure to high metal and its uptake to the shoot by restricting their accumulation to the root. Heavy metal hyperaccumulators that accumulate metals preferentially transport them to the above-ground parts (Jaffré et al., 1976; Pollard et al., 2002).

Until now, more than 450 plant species have been characterized from a number of different families such as the Asteraceace, Brassicaceae, Caryophyllaceae, Poaceae, Violaceae, and Fabaceae and most of them are nickel (Ni) tolerant (Verbruggen et al., 2009a; Krämer, 2010). Among these hyperaccumulators, only three plant species are classified to be Cd hyperaccumulators: *Arabidopsis halleri* (Bert et al., 2002), *Noccaea caerulescens* (Brooks et al., 1977; Baker et al., 1994) (formerly *Thlaspi caerulescens*) and *Sedum alfredii* Hance (Yang et al., 2004). At the forefront of studies concerning the hyperaccumulation process *T. caerulescens* and *N. caerulescens* have been used (Milner and Kochian, 2008). Due to the relatively low biomass and slow growth, they primarily served as model systems to investigate and identify the underlying molecular and physiological mechanisms of heavy metal hyperaccumulation. Ultimately the research aims is to apply the knowledge gained about these mechanisms to improve higher biomass of crop plants (Verbruggen et al., 2009a; Krämer, 2010). Their potential for use in decontamination of large-scale Cd-contaminated soils is not practically applicable. Moreover, in recent years, numerous studies have been focused on determining the feasibility of applying the mechanisms that metal-hyperaccumulator plant species employ in the breeding of plants suited for the remediation or phytoremediation of metal-contaminated soils. Therefore, it is essential to investigate hyperaccumulation mechanisms of other Cd hyperaccumulators, particularly non-Brassica species with potential practical applications.

The hyperaccumulating ecotype (HE) of *Sedum alfredii* Hance is a Zn/Cd co-hyperaccumulator originally found in a region of China with soils rich in PB/Zn, possessing remarkable traits of accumulating up to 9,000 μg g^-1^ Cd and 29,000 μg g^-1^ Zn in its shoots without any toxicity symptoms (Yang et al., 2002, 2004). It is a species native to China and the only one metal hyperaccumulator plant from the Crassulaceae family. The potential of *S. alfredii* Hance for practical decontamination is related to its large biomass, rapid growth, and asexual propagation. Also it could serve as an ideal candidate for obtaining a functional understanding of the intra-family hyperaccumulation mechanisms (Sun et al., 2009). As a species not been studied extensively and profoundly, the underlying mechanisms for hyper-tolerance in *S. alfredii* Hance (HE) is poorly elucidated, due in part to the lack of genes identified to be involved in heavy metal resistance or accumulation.

Based on our previous studies, we screened for functional genes related to Cd tolerance through expressing cDNA library in yeasts and obtained a gene resembling the *rubber elongation factor* (*REF*) gene. REF, previously identified in rubber biosynthesis from *Hevea brasiliensis* and termed by Dennis and Light (1989), is an abundant protein associated with the lipid membrane surrounding the rubber particles. Previous studies have found that this protein is an essential element required for rubber molecule elongation which interacts with prenyl transferase to alter the stereochemistry of isopentenyl pyrophosphate (IPP) addition from the normal *trans* addition to *cis* and overrides the normal termination after two *trans* additions to affect the formation of *cis*-polyisoprene (Dennis et al., 1989). Although proteins belonging to a larger stress-related protein (SRP) family from plants, and several SRPP non-latex producing homologous genes and genes containing REF domains have been reported to be involved in stress response (Qi et al., 2009), direct evidences confirming REF or SRPP as SRPs were still deficient (Berthelot et al., 2014). Most findings until present are embracing the context of their roles in rubber biosynthesis while functions in other processes such as stress response are seldom reported (Kush et al., 1990; Han et al., 2000; Aoki et al., 2014).

In our previous study, a gene containing the *REF* domain in *S. alfredii* Hance (HE) was detected to improve yeast tolerance to cadmium. Herein, we performed further studies to validate and elucidate functions of this gene in cadmium resistance in *S. alfredii* Hance (HE). The enhanced expression of this gene may contribute to Cd hyperaccumulation and hypertolerance in *S. alfredii* Hance.

## Materials and Methods

### Plants Materials and Growth Condition

Hyperaccumulating ecotype of *S. alfredii* Hance (HE), first identified by Yang et al. (2002), was found to be flourishing in the deserted Pb/Zn mining district located in Quzhou city, Zhejiang Province, PR China. *S. alfredii* Hance (HE) seedlings were cultured in buckets filled with tap water and exposed to 25°C and long days (16 h light/8 h dark each day) in an artificial climate growth chamber. The *S. alfredii* seedlings for the stress treatment were vegetatively propagated to ensure homogeneity and grown in half-strength Hoagland–Arnon solution (Hoagland and Arnon, 1950) for about 2 weeks until the relatively vigorous roots grew out.

*Arabidopsis thaliana* (ecotype Col-0) plants were grown on potted soil in a climate-controlled greenhouse under controlled environments (16 h light/8 h dark cycles, white light intensity approximately 125 μmol m^-2^ s^-1^ at 22°C and 50% relative humidity) till plant completed its life cycle. In experiments that included homozygous transgenic plants (T3), seeds were surface sterilized and germinated on 1/2 MS (Murashige and Skoog, 1962) agar plates containing 25 mg L^-1^ hygromycin. Three-week-old wild-type, homozygous transgenic seedlings were used in Cd resistance and other experiments.

### Cloning and Sequence Analysis of *SaREFl*

In order to identify *S. alfredii* Hance (HE) genes that confer Cd tolerance, we constructed a cDNA library of *S. alfredii* Hance (HE) with a SMART cDNA Library Construction Kit (CLONTECH, Palo Alto, CA, USA) according to the recommended protocol and transformed the library into the INV*Sc*1 yeast strain. This strain does not grow on half-strength synthetic galactose agar plates lacking uracil and containing 80 μM CdCl_2_. We selected transformed colonies that grew in the presence of 80 μM CdCl_2_ and then isolated and sequenced the respective plasmids.

Among these colonies, there was a sequence of interest encoding a 248 amino acid polypeptide with homology to REF which had rarely been reported to be related to heavy-metal stress. The sequence was analyzed for coding probability with the DNATools program (Rehm, 2001). Comparison against the GenBank protein database was performed using the BLAST network server at the National Center for Biotechnology Information (Altschul et al., 1997), based on which the sequence was designated as *S. alfredii* Hance (HE) *REF like* gene (*SaREFl*). Multiple protein sequences representing the homologous genes from other species with the Genbank accession numbers were listed in **Table 1**. Homology of SaREFl with the REF family proteins was aligned using the MegAlign program by the CLUSTALW method in the DNASTAR software package (Burland, 2000). A phylogenetic tree was constructed using the neighbor-joining method within the package PHYLIP 3.5c software package (Felsenstein, 1993) using 1000 bootstrap replicates.

**Table 1:** Names and accession numbers of the REF protein family.

Species	Name	Accession numbers
*Sedum alfredii*	SaREF	KP771801
*Medicago truncatula*	MtREF	KEH20598
*Arabidopsis thaliana*	AtREF	NP_187201.1
*Copaifera officinalis*	CoREF	AEX97052.1
*Populus trichocarpa*	PtREF	XP_006382485.1
*Brassica campestris*	BcREF	AHY19029
*Ricinus communis*	RcREF	XP_00251247.1
*Theobroma cacao*	TcREF1	XP_007030996.1
*Theobroma cacao*	TcREF2	XP_007030997.1
*Citrus sinensis*	CsREF	XP_006472088
*Morus alba*	MaREF	ACV90044.1
*Hevea brasiliensis*	HbREF1	P15252
*Hevea brasiliensis*	HbREF2	AEH05970
*Hevea brasiliensis*	HbREF3	AAR11448
*Taraxacum brevicorniculatum*	TbSRPP1	AGE89406
*Taraxacum brevicorniculatum*	TbSRPP2	AGE89407
*Taraxacum brevicorniculatum*	TbSRPP3	AGE89408
*Taraxacum brevicorniculatum*	TbSRPP4	AGE89409
*Hevea brasiliensis*	HbSRPP	AGO95096.1
*Ricinus communis*	RcSRPP	XP_002531884
*Nelumbo nucifera*	NnSRP	XP_010259606.1
*Oryza brachyantha*	ObSRP	XP_006658100
*Phoenix dactylifera*	PdSRP	XP_008786716
*Nicotiana tomentosiformis*	NtSRP	XP_009602185
*Nicotiana sylvestris*	NsSRP	XP_009781897
*Solanum tuberosum*	StSRP	XP_006359392.1
*Solanum lycopersicum*	SlSRP	XP_004247432
*Eucalyptus grandis*	EgSRP	XP_010038197
*Ipomoea batatas*	IbSRP	ABP35522
*Cicer arietinum*	CaSRP	XP_004485771
*Citrus sinensis*	CsSRP	XP_006479926
*Malus domestica*	MdSRP	XP_008386654
*Prunus mume*	PmSRP	XP_008235050.1
*Fragaria vesca*	FvSRP	XP_004290778

**FIGURE 1:**
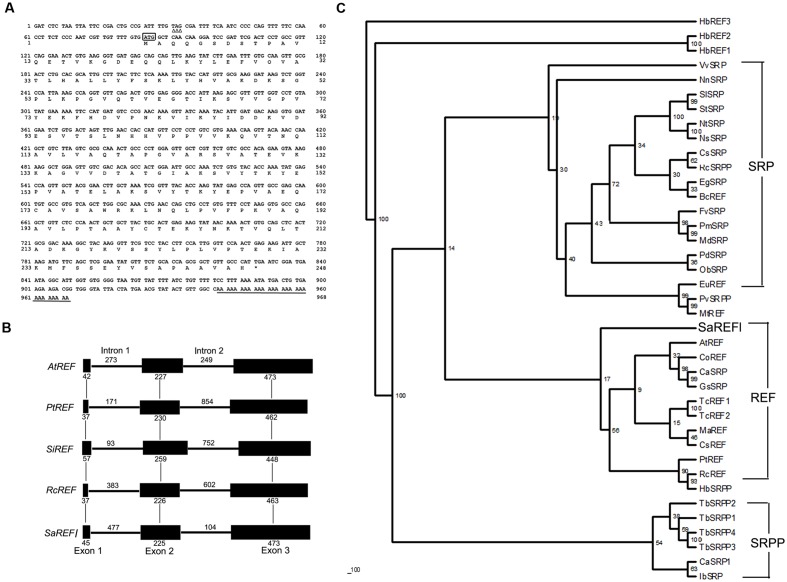
**Bioinformatics analysis of *SaREFl*. (A)** Nucleotide and deduced amino acid sequences of *Sedum alfredii REFl* cDNA (accession number in GenBank: KP771801). The in-frame stop codon in 5′UTR is marked by Δ. The start codon is boxed. The asterisk represents the stop codon. The polyadenylation signal is underlined by a single line. **(B)** Phylogenetic trees of *SaREFl* with other representative members of *REF* protein family. The phylogenetic trees of *SaREFl* as well as their counterparts of other REFs and REF protein family members constructed by the neighbor-joining method within the package PHYLIP 3.5c. Bootstrap majority consensus values on 1000 replicates are indicated at each branch point in percent. Accession numbers for sequences used are listed in **Table 1**. **(C)** Diagram of the genomic structures of *REF* genes from *Sedum alfredii*, *Populus trichocarpa*, *Seteria italic*, *Arabidopsis thaliana*, and *Ricinus communis*. Solid boxes represent the region coding for the structural sequences of the protein. Thin lines indicate the sequences of introns. The number of nucleotides above the thin lines represent the size of the introns, and the number below the solid boxes the size of the exons. The genomic sequences used in the analysis were listed as follows: *Populus trichocarpa REF* (*PtREF*, POPTR_0013s01800g), *Seteria italic REF* (*SiREF*, NW_004675963.1), *Arabidopsis thaliana REF* (*AtREF*, AT3G05500F22F7.5), and *Ricinus communis REF* (*RcREF*, RCOM_1433280).

**FIGURE 2:**
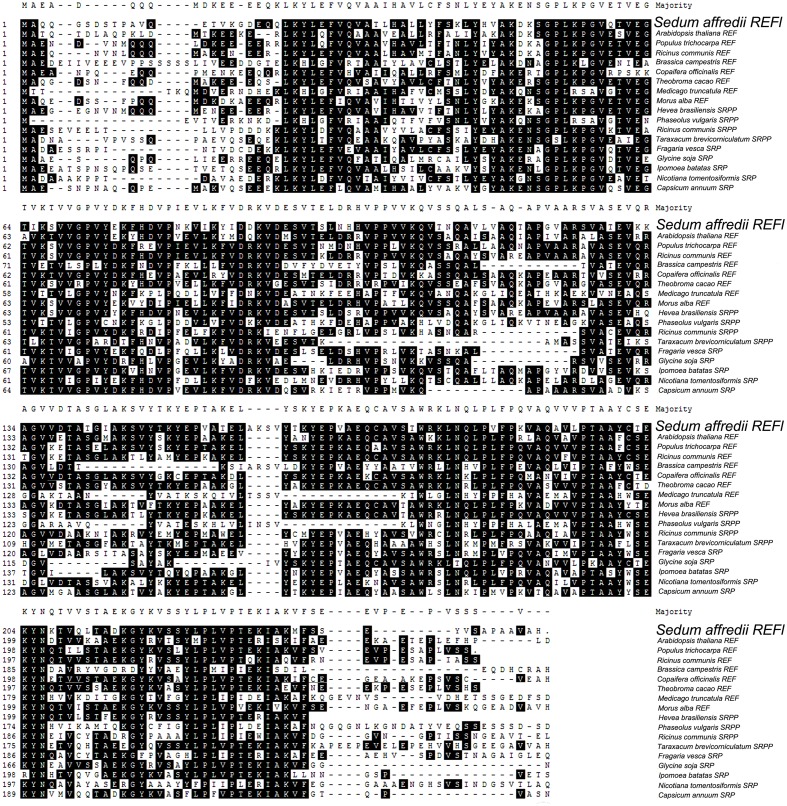
**Sequences of SaREFl and proteins belonging to REF family were aligned using the MegAlign program by the CLUSTAL method in the DNASTAR software package.** Identical residues are shaded in black and gaps were introduced into sequences to optimize alignment. GenBank accession numbers of the 18 amino acid sequences were listed in **Table 1**.

*See **Figure 1** for the phylogenetic trees and **Figure 2** for the alignment of amino acid sequence. REF, rubber elongation factor; SRPP, small rubber particle protein; SRP, stress-related protein.*

Moreover, the genomic sequence of *SaREFl* was amplified through PCR using genomic DNA as templates with primers SaREFO-F and SaREFO-R listed in **Table 2**. In order to compare the genomic structure, we searched the genomic sequences of *Populus trichocarpa REF* (*PtREF*, POPTR_0013s01800g), *Seteria italic REF* (*SiREF*, NW_004675963.1), *Arabidopsis thaliana REF* (*AtREF*, AT3G05500F22F7.5) and *Ricinus communis REF* (*RcREF*, RCOM_1433280) from GenBank and analyzed the intron-exon structure.

**Table 2:** Primers designed for the experiments in the study.

Primer name	Primer sequence (5′–3′)	Sequence information
*GAL1*- Forward (sense)	5′-AATATACCTCTATACTTTAACGTC-3′	Validation and sequencing primer for pYES2.1-SaREFl
V5 C-term Reverse (antisense)	5′-ACCGAGGAGAGGGTTAGGGAT-3′	Validation and sequencing primer for pYES2.1-SaREFl
pENTR-F (sense)	5′-CACCATGGCTCAACAAGGATCCG-3′	Construction of Gateway entry clone
pENTR-R (antisense)	5′-TCAATGGGCAACAGCCGC-3′	Construction of Gateway entry clone
SaREF-F (sense)	5′-TGCCGAGCAATGTGCCGTGTC-3′	Primers for real-time PCR
SaREF-R (antisense)	5′-CCTTGTAGCCTTTGTCCGCAGTGAG-3′	Primers for real-time PCR
TUB-F (sense)	5′-TTATGGCGATTCCGAGCTTCA-3′	Primers for real-time PCR
TUB-R (antisense)	5′-ATTATTTCCAGCGCCGCATTG-3′	Primers for real-time PCR
Hyg-F (sense)	5′-GTTTATCGGCACTTTGCATCG-3′	Validation of transgenic *Arabidopsis*
Hyg-R (antisense)	5′-GGAGCATACGCCCGGAGT-3′	Validation of transgenic *Arabidopsis*
SaREFO-F (sense)	5′-ATGGCTCAACAAGGATCCG-3′	Validation of transgenic *Arabidopsis*
SaREFO-R (antisense)	5′-TCAATGGGCAACAGCCGC-3′	Validation of transgenic *Arabidopsis*

### Assays for Effects of Different Heavy-Metal Ions on Expression of *SaREFl*

In order to validate the expression profiles of *SaREFl* under heavy-metal stress, *S. alfredii* Hance (HE) seedlings were subjected to different heavy-metal ions treatment and the expression profile of *SaREFl* was examined by means of qRT-PCR. The *S. alfredii* Hance (HE) seedlings were obtained by asexual propagation. We cut the shoot tops of *S. alfredii* Hance (HE), grew them further in a greenhouse for 2 months, and then vigorous and uniform seedlings were selected for use. The selected seedlings were transferred to half-strength Hoagland–Arnon solution (pH 6.0) which was aerated continuously and renewed every 2 days. After 2 weeks of growth, the seedlings with relatively flourishing roots were randomly divided into experimental and control groups in a study on the effects of heavy-metal stress. The concentrations of the heavy metals were set according to a previous study characterizing the expression of a reliable internal gene for *S. alfredii* Hance (Sang et al., 2013). Briefly, the experimental groups were treated with 400 μM CdCl_2_, 400 μM Pb(NO_3_)_2_, 500 μM CuSO_4_ and 500 μM ZnSO_4_, respectively, while the control ones were cultured in water.

Three different plant organs (roots, stems, and leaves) were collected at different time points including 0, 0.5, 6, 12, 24, 48, and 72 h. The respective organs of the seedlings in the control group were also sampled. All experiments were performed in triplicates. All plant materials collected were promptly frozen in liquid nitrogen and kept at -80°C until total RNA extraction.

Total RNA was prepared according to the manual of the Total RNA Purification Kit (Norgan Biotek Corp., Thorold, ON, Canada) and then treated with RNase-free DNase I (NEB BioLabs, Ipswich, MA, USA) to remove potential genomic DNA contamination. The quality of total RNA was first assessed by agarose gel electrophoresis and then further quantified by a NanoDrop2000 spectrophotometer (Thermo Scientific, Wilmington, DE, USA). The first strand cDNA was generated from 3 μg of RNA sample with the SuperScript III First-Strand Synthesis System (Invitrogen, Carlsbad, CA, USA) using oligo d(T) primer followed by the RNase H step (Invitrogen, Carlsbad, CA, USA). Following quantification of the cDNA templates and examination of primers (*SaREF*-F/*SaREF*-R, **Table 2**), qRT-PCR was performed on ABI 7300 real-time PCR system (Applied Biosystems, USA) as described by Liu et al. (2014). The housekeeping *TUB* gene was used as the reference for internal standardization validated by Sang et al. (2013) and the detailed primer sequences (TUB-F/TUB-R) were listed in **Table 2**.

### Cloning and Construction of Expression Vectors

To assess Cd tolerance in yeast cells, purified full-length *SaREFl* products without stop codon were cloned into the pYES2.1/V5-His-TOPO vector (Invitrogen, Carlsbad, CA, USA) and positive clones were sequenced by general primers *GAL1* Forward and V5 C-term Reverse sequencing primers provided by the kit (**Table 2**).

For the gene overexpressing and subcellular location in *Arabidopsis*, the cDNA of *SaREFl* was also amplified by PCR using High Fidelity KOD-Plus DNA Polymerase (Toyobo, Japan) with primers pENTR-F and pENTR-R listed in **Table 2**. The purified PCR products were then cloned into the Gateway entry vector pENTR and positive clones were further sequenced to verify the direction and sequence accuracy. The verified plasmid was then recombined in pH2GW7 and pK7WGF2.0 to generate pH2GW7-*SaREFl* for overexpression and pK7WGF2.0-*SaREFl* for determination of their subcellular locations, respectively.

All the constructs above were verified by sequencing prior to use to confirm error- free amplification and cloning. Primers used during the construction and their uses for preparation of specific constructs were listed in **Table 2**.

### Heterologous Expression of *SaREFl* and Cd Tolerance test in *Saccharomyces cerevisiae*

To assess Cd tolerance in yeast cells, the correct pYES2.1/V5-His-TOPO construct was transformed into Cd-sensitive mutant yeast strain DTY167 (MATa ura3-52 his6 leu2-3,-112 his3-D200 trp1-901 lys2-801 suc2-D, ycf1::hisG) by the lithium acetate method (Szczypka et al., 1994). Transformed yeast cells were selected on half-strength synthetic dextrose agar plates lacking uracil.

For assessment of growth inhibition in response to Cd, *SaREFl* expressing yeast lines were grown up to 2 OD_600__nm_ units followed by serial dilution and spotting on half-strength synthetic galactose-uracil (SG-ura) agar plates in the absence or presence of 30 μM CdCl_2_ at 30°C for 3 days before photographs were taken. To examine the relative growth inhibition, yeast cultures on logarithmic phase were firstly diluted to OD_600nm_ of approximately 0.4 unit with half-strength SG-ura supplemented with 30 μM CdCl_2_, and then the yeast cultures were kept on growing at 30°C for 72 h. Relative growth was determined through measuring OD_600__nm_ every 12 h. Empty vector (EV) pYES2.1/V5-His-TOPO transformed yeast cells were considered as control in each experiment.

To determine whether expression of *SaREFl* conferred tolerance to other heavy metals, the constructed pYES2.1/V5-His-TOPO vector containing *SaREFl* was introduced into yeast strain INVSc1 using a lithium acetate method (Szczypka et al., 1994). Two lines of transformed yeast cells containing the vectors pYES2.1/V5-His-TOPO (control) or pYES2.1-*SaREFl* were cultivated on medium supplemented with 5 mM Cu^2+^, 1.5 mM Pb^2+^ and 15 mM Zn^2+^ at 30°C for 3 days before being photographed. The concentrations of the above three heavy metals were set according to a previous study characterizing the functions of a heavy metal-associated protein (*AcHMA1*) from the Halophyte, *Atriplex canescens* (Pursh) Nutt. (Sun et al., 2014).

### Overexpression of *SaREFl* in *Arabidopsis* and Cadmium Stress Treatment

*Arabidopsis thaliana* Columbia was transformed with the plasmid pH2GW7-*SaREFl* with the *Agrobacterium*-mediated floral dip method as described previously (Hsiao et al., 2007). Several independent lines (T1) were selected on 1/2 MS agar plates containing 25 mg L^-1^ hygromycin and transgenic lines were further validated through PCR. The PCR validation was based on genomic DNA template primed with general primers of antibiotic (Hyg-F/Hyg-R) and specific gene primers (SaREFO-F/SaREFO-R) listed in **Table 2**. The expression of *SaREFl* in transgenic *A. thaliana* was asserted by RT-PCR using specific gene primers (SaREFO-F/SaREFO-R). Total RNA was extracted from young leaves of transgenic and WT plants and first strand cDNA was synthesized as described above. RT-PCR was carried out with parameters set as below: denaturation at 94°C for 5 min, 30 cycles of amplifications (94°C for 30 s, 55°C for 30 s, and 72°C for 60 s), followed by a final cycle of 72°C for 7 min. Three T3 homozygous lines (OE-1, OE-2, and OE-3) with single copy insertions were used to analyze the growth characteristics under normal and heavy-metal stress conditions in the following tolerance study.

For root length measurements, seeds of WT and three transgenic *A. thaliana* homozygous lines expressing *SaREFl* were germinated and grown vertically on half-strength MS (½ MS) media in the presence or absence of 100 μM cadmium for 10 d after stratification (4°C for 48 h in dark) (Thomine et al., 2003). After the indicated days of growth, *A. thaliana* seedlings were spread on a filter paper and individual root length was determined using a vernier caliper. The fresh weight (FW) was subsequently weighed and recorded. The experiments were replicated three times. The variables were expressed as mean standard deviation (SD) of data collected for 15–20 independent plants for each line. Statistical differences between WT and three transgenic lines were calculated by student *t*-test (*P* < 0.05).

For adult plant experiments, four-leaf stage wild-type and *SaREFl*-expressing *Arabidopsis* seedlings grown on MS agar were replanted into potted soils and grown under a 16/8-h photoperiod at 22°C for 3 weeks. Then the plants were watered with 500 μM CdCl_2_ and sampled 10 days later. Healthy young leaves of stress-treated and control plants were taken for further physio-biochemical analyses. Lipid peroxidation was determined based on the concentration of malondialdehyde (MDA) produced by thiobarbituric acid (TBA) reaction (Draper and Hadley, 1990). In brief, leaf samples were extracted in 2 ml 0.1% trichloroacetic acid (TCA) and 0.5 ml leaf extract was reacted with 2.0 ml of TBA reagent followed by boiling at 95°C for 30 min. The reaction mixtures were cooled on ice, centrifuged at 10,000 *g* for 5 min and absorbance of the supernatants was measured at 440, 532, and 600 nm.

To determine electrolyte leakage (EL), leaves were firstly washed thoroughly with deionized water to remove surface-adhered electrolytes, and then they were kept in closed vials containing 10 ml of deionised water and incubated at 25°C on a rotary shaker for 24 h. Subsequently, the electrical conductivity (EC) of the solution (L_t_) was determined using a conductivity meter (SevenEasy, Mettler Toledo AG 8603, Switzerland). Leaf extracts were autoclaved at 120°C for 20 min, followed by cooling to 25°C and then EC (L_0_) was determined. The EL was determined: EL (%) = (L_t_/L_0_) X 100.

Cadmium content measurements were also performed. Stress-treated plants were washed in distilled water three times and desorbed for 10 min with 2 mM CaSO_4_ and 10 mM ethylenediaminetetraacetic acid to remove surface and apoplastic heavy metals, then the shoots and leaves were excised from the plants, followed by the drying at 105°C for 30 min and 80°C for 48 h (Cailliatte et al., 2010). After weighing, the dry tissues were ground into powder and then digested in HNO_3_/HClO_4_ (4:1, v/v). The acid digests were diluted with deionized water and Cd^2+^ concentration was determined using atomic absorption spectrophotometry (Perkin-Elmer model 3300). The experiment was replicated three times, with each replicate consisting of at least 12 plants.

### Yeast Two-Hybrid Experiments

In order to identify potential genes interacting with *SaREFl*, the yeast two-hybrid experiments using yeast mating were carried out following the instructions supplied by Clontech Matchmaker Gold Yeast Two-Hybrid System. Firstly, for the preparation of cDNA library, total RNA was extracted from fresh tissues including root, stem, and leaf using an RNeasy Plant Minikit (Qiagen) and treated with DNase I (Ambion). Matchmaker Library Construction and Screening kit (Clontech, Mountain View, CA, USA) was applied to generate an activation domain (AD)-tagged cDNA library, which was subsequently cloned into the plasmid pGADT7-sfiI (~2.5 × 10^6^ clones). The mixed plasmids of positive inserts were further amplified in yeast strain Y187. A *GAL4* DNA-BD fusion was generated by cloning *SaREFl* in frame with the *GAL4* DNA binding domain of the bait plasmid pGBKT7 and transformed into cells of the yeast strain Y2HGold. It is imperative to confirm that the bait *SaREFl* does not autonomously activate the reporter genes in Y2HGold, in the absence of a prey protein. The absence of self-activation was verified by transformation of the bait alone, selected on minimal medium lacking tryptophan and histidine.

For library screening, the Clontech Matchmaker Gold Yeast Two-Hybrid System was applied to perform the yeast two-hybrid experiments using yeast mating. A concentrated overnight culture of the bait strain (Y2HGold [pGBKT7 + *SaREFl*]) was combined with the library strain and incubated at 30°C for approximately 20 h with gentle shaking until zygotes could be observed under a phase contrast microscope (40X). The positive colonies that activated at least two reporter genes (His 3, Ade2 or LacZ) were plated onto new plates and further identified by sequencing. In order to confirm that the positive interactions are genuine, the library plasmid responsible for activation of reporters was rescued and then co-transformation of pGBKT7/*SaREFl* and positive AD/library plasmids into Y2HGold was conducted. Those independent colonies that could activate two reporter genes (His 3, Ade2) were preliminarily verified as being genuine and the prey insert could be identified by sequencing. Y2HGold [pGBKT7-53] mating with Y187 [pGADT7-T] was applied as positive control. Negative control was performed using pGBKT7-Lam and pGADT7-T. All control constructs were provided by the manufacturer (Clontech).

### Transient Expression and Subcellular Localization of *SaREFl*-GFP

The verified construct of pK7WGF2.0-*SaREFl* for subcellular localization study was used to transform onion epidermal cell layers using Helium Biolistic gene transformation system (Du Pont). Inner epidermal layers were peeled and placed inside up on Petri dishes containing MS basal medium containing 2.5 mg L^-1^ amphotericin B (Sigma, St. Louis, MO, USA) and 6% (w/v) agar. DNA samples containing pK7WGF2.0-*SaREFl* were prepared as described by the manufacturer (Du Pont) which were coated with 1 μM gold particles and followed by the introduction into onion epidermal cells using particle bombardment (PDS-100/He particle delivery system, Bio-rad). Then, Petri dishes were sealed with parafilm and incubated for 18–24 h at 28°C in the dark. After 20 h, fluorescent cells were imagined. The green fluorescence was observed through a laser scanning confocal microscope (LSM510; Karl Zeiss, Jena, Germany) excitated at 488 nm for GFP.

### Statistical Analysis

Each experiment was performed under a completely randomized design with independent experiments with at least three replications. Level of significance was calculated using student *t*-test (*P* < 0.05).

## Results

### Sequence and Phylogenetic Analysis of *SaREFl*

To identify *S. alfredii* Hance (HE) genes that confer Cd tolerance, we transformed the yeast strain INV*Sc*1 with a *S. alfredii* cDNA library. We selected colonies that grew in the presence of 80 μM CdCl_2_ and sequenced the respective plasmids. Among these colonies, a transcript of interest displayed a high homology to REF. The BLASTp searching at NCBI showed that the protein encoded by the cDNA that has a REF domain at residues 11–239. Therefore, the cDNA encoded an *S. alfredii* Hance (HE) *REF-like* gene, and we designated it as *SaREFl*. REF was a protein named in the context of their roles in rubber biosynthesis and had received the most attention because they were the most abundant rubber membrane particles. Interestingly, it had rarely been reported to be related to heavy-metal stress. Multiple analysis tools were applied to uncover the preliminary bioinformatics features of *SaREFl*.

The full-length cDNA of *SaREFl* is 968 bp, and its longest open reading frame codes for a protein of 248 amino acids with a predicted molecular mass of approximately 26.97 kDa. The 5′-untranslated region (UTR) is 78 bp, and the 3′-UTR is 137 bp with a polyadenylation tail (**Figure 1A**). The initiation codon (ATG) was assigned on the basis that there is no ATG in the 5′-UTR 78 nucleotides and the DNA surrounding the initiation codon ATG has a purine at positions of both -3 and +4, which is in accordance with the Kozak consensus sequence (A/GXXATGG) and is suggested to optimize translational efficiency (Kozak, 1987). SignalP software analysis by the Signal IP 3.0 server (Nielsen et al., 1997) revealed that *SaREFl* had no putative N-terminal signal peptide. The *SaREFl* sequence was submitted to the GenBank database and assigned with an accession number KP771801.

Rubber elongation factor belongs to the REF family which is classified into three highly related categories including *REF*, small rubber particle protein (*SRPP*) and *SRP*. Like other REFs, SaREFl was similar in terms of structure and characterized by the presence of REF domain covering approximately 225 amino acids. A phylogenetic tree was constructed using the amino acid sequence of *SaREFl* as well as the counterparts of other representative *REFs* and REF family members to investigate the relationship among them. Basically, the homologous proteins analyzed were classified into three major groups (REF, SRP and SRPP) although several exceptions existed. SaREFl was clustered together with other REFs being obviously distinct from SRP and SRPP classes and located separately at the base of the subfamily containing most REF proteins (**Figure 1B**).

Analysis of the genomic structure exhibited that *SaREFl* gene consisted of three exons and two introns which was in accordance with findings of *Hevea brasiliensis* REF (Priya et al., 2006). The three exons of 45, 255, and 473 bp, respectively, were interspaced by two introns of 477 and 104 bp, which all began with GT and ended with an AG dinucleotide (**Figure 1C**). These sequences have been thought to be necessary for correct RNA splicing of various other eukaryotic genes (Breathnach et al., 1978). It was notable that the *Populus trichocarpa*, *Seteria italic*, *Arabidopsis thaliana*, and *Ricinus communis REF* genes all had three exons and two introns, indicating that *SaREFl* gene had an exon-intron organization similar to that of representative species of woody and herbaceous plants.

Further comparison with representative members of REF family demonstrated that SaREFl was much more closely identical to REFs comparing with that of SRPs and SRPPs. Among REFs, SaREFl shared with 61.8, 61.3, 61.2, and 61% identity to *REFs* of *R. communis*, *Populus trichocarpa*, *Theobroma cacao* and *Morus alba*, respectively, while it had less than 40% identity to the REFs of *Brassica campestris* and *Medicago truncatula* (**Figure 2**).

### Transcript Analysis of *SaREFl* under Different Heavy-Metal Stress

**FIGURE 3:**
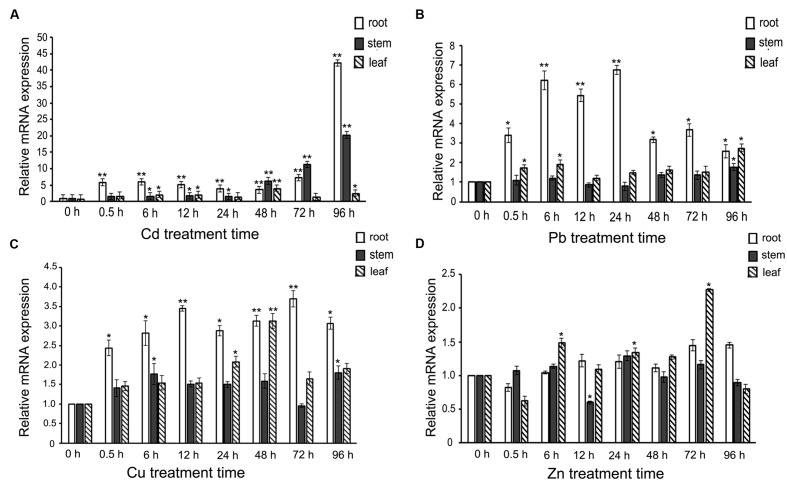
**Expression profiles of *SaREFl* upon treatment of *Sedum alfredii* seedlings with four heavy-metal ions (Cd, Pb, Cu, Zn) for different amounts of time. (A)** Real-time qPCR analysis of *SaREFl* expression level under cadmium stress. **(B)** The effect of Pb treatment on *SaREFl* expression. **(C)** Expression profile of *SaREFl* under Cu treatment. **(D)** Changes of *SaREFl* transcript level under Zn stress. The time of exposure is indicated on the *x*-axis; the *y*-axis indicates the mRNA expression relative to *TUB* which was selected as an internal reference gene. The normalized mRNA levels without treatment (*y*-axis “Relative mRNA expression”) were set arbitrarily to 1. Vertical bars represent means ± SD (*n* = 3). Student’s *t*-tests was applied to evaluate statistical significance and significant differences (*P* < 0.05 and *P* < 0.01) are indicated by * and **respectively.

When *S. alfredii* Hance (HE) seedlings were challenged with heavy-metal ions, how would *SaREFl* react? To answer this question, the expression profiles of *SaREFl* were investigated by a time-course treatment containing eight time points with four heavy metals. Among the four heavy-metal ions (Cd, Zn, Cu, Pb), we found that *SaREFl* reacted more dramatically to Cd treatment while no obvious up-regulation was observed under Zn treatment (**Figure 3**). When comparing the transcript levels in root, stem, and leaf under Cd excess, *SaREFl* showed a higher sensitivity in roots than other tissues, displaying a large-scale elevation of expression from the very beginning, maintaining the trend and reaching the peak at 96 h (**Figure 3A**). A similar trend was detected in the stems, with a high level of the transcript accumulation from 48 h and keeping rising until the treatment ended (**Figure 3A**). In the leaves, a relatively steady expression profile was found (**Figure 3A**).

When the plants were treated with Pb, the expression of *SaREFl* was up-regulated instantly in the roots and the expression summit occurred at 24 h, then returned back to the level of 0.5 h at 48h and 96 h after treatment (**Figure 3B**). No dramatic response was detected in the tissues of stem except at 96 h (**Figure 3B**) and the induced level in leaf showed slight elevation just at 0.5, 6 and 96 h (**Figure 3B**). The profile of *SaREFl* responding to Cu stress was basically similar to that under Pb stress with divergences mainly occurring in the responding time points and amplitude of variation (**Figure 3C**). Interestingly, although *S. alfredii* was characterized with Zn/Cd hyper-accumulating traits, *SaREFl* demonstrated a steady-state expression to Zn stress except at three time points in the leaf (**Figure 3D**).

Based on the above results, we found that the expression of *SaREFl* was induced by heavy metals and divergent patterns of responses were observed among different metal irons.

### *SaREFl* Enhances Cd Tolerance in the Yeast Assays

**FIGURE 4:**
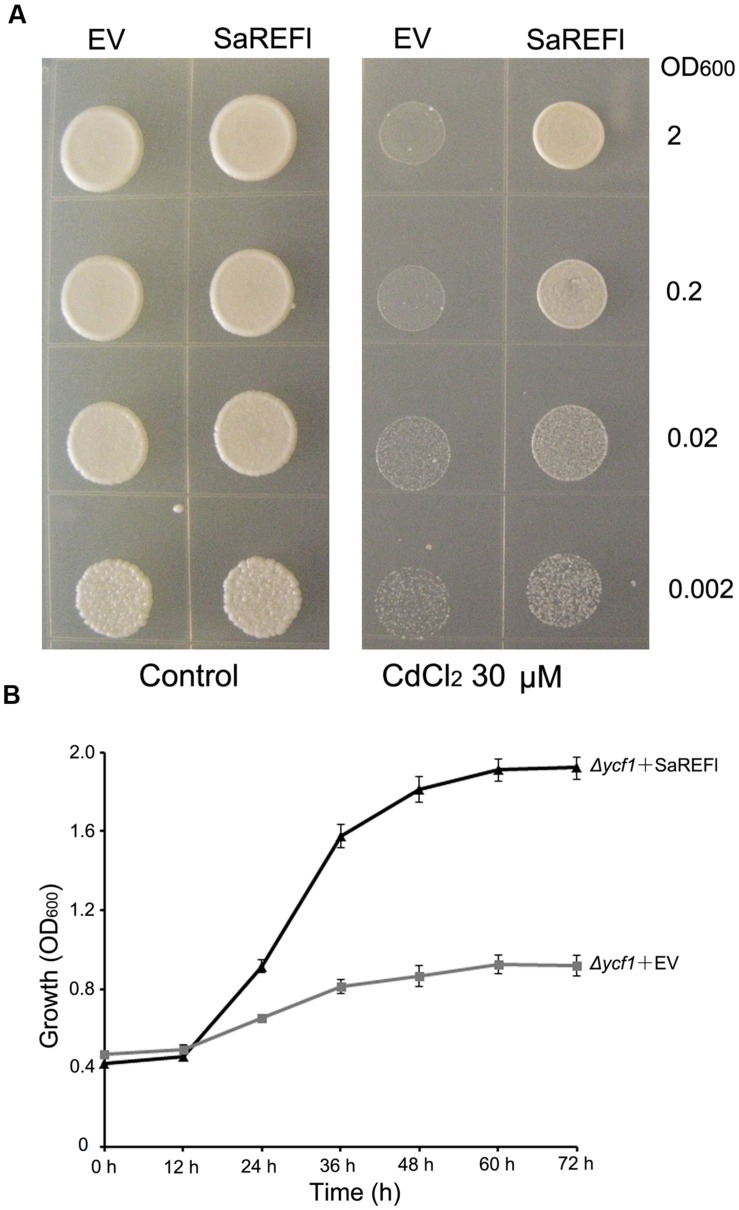
**Verification of cadmium tolerance of *SaREFl* in yeast. (A)** Exhibition of *SaREFl*-conferred cadmium resistance in the *Δycf1* yeast mutant. Expression of *SaREFl* brought about enhanced growth of *SaREFl*-transformed yeast on half-strength SG-ura agar plates supplemented with 30 μM CdCl_2_ compared with yeast strains transformed with empty vectors. OD600, optical density of the yeast suspension at 600 nm. **(B)** Time-dependent growth of yeast strains in SG-ura liquid medium supplemented with 30 μM CdCl_2_. The data are means and SD from three independent experiments.

To verify the capability of *SaREFl* to rescue Δ*ycf1* phenotype, yeast expression vector pYES2.1 containing *SaREFl* was transformed into Δ*ycf1*, which does not grow in medium containing 60–80 μM CdCl_2_ due to the defect of Cd sequestration into the vacuole (Shim et al., 2009). In addition, the EV pYES2.1 transformed yeast cells were considered as control in each experiment. Both Δ*ycf1* strains carrying the *SaREFl* expression vector and EV pYES2.1 grew well under non-stressed conditions. In contrast, on half-strength SG agar medium supplemented with 30 μM CdCl_2_, the Δ*ycf1* mutant transformed with pYES2.1-*SaREFl* construct was able to survive at different dilutions whereas growth of control was arrested (**Figure 4A**). Similarly, in SG-ura liquid medium supplemented with 30 μM CdCl_2_, Δ*ycf1* cells expressing *SaREFl* exhibited dramatically enhanced growth when compared with Δ*ycf1* cells transformed with the EV (**Figure 4B**). Moreover, transformed INVSc1 yeast cells expressing *SaREFl* were applied to test the tolerance to Zn^2+^, Cu^2+^ and Pb^2+^. No significant differences were observed comparing the growth of yeast strain carrying empty vector with that of yeast cells transformed with pYES2.1-*SaREFl* (**Supplementary Figure S1**). These observations suggest that *SaREFl* was able to rescue inability of Δ*ycf1* to grow in medium containing a certain amount of cadmium which shows its relevance for cadmium tolerance.

### Transgenic *SaREFl* Expressing *Arabidopsis* Lines Displayed Cd Tolerance and Accumulation

**FIGURE 5:**
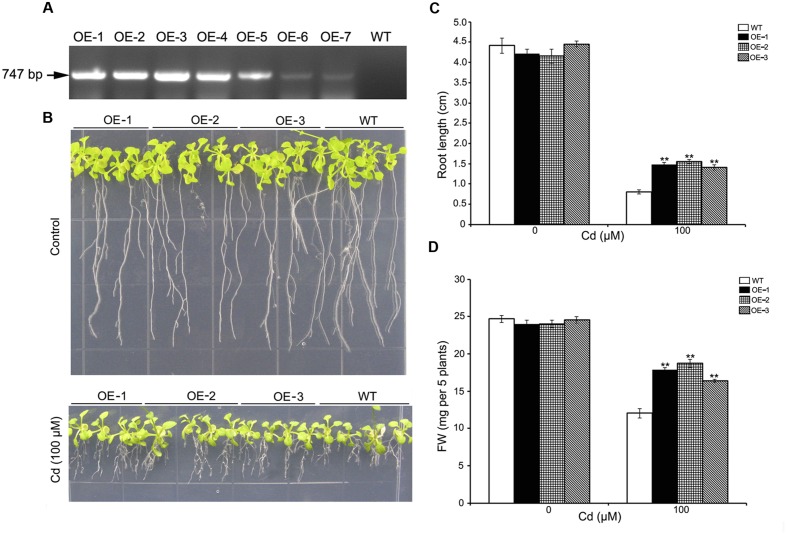
**Expression of *SaREFl* in *Arabidopsis* confers tolerance for Cd. (A)** RT-PCR detected the expression of *SaREFl* in transgenic plants. **(B)** Phenotypic changes of WT and transgenic lines (OE-1, OE-2, and OE-3) grown vertically for 10 days on ½ MS media with or without cadmium. **(C,D)** Root length and fresh weight of plants described in **(B)**. Bars represent the mean of root length of 15–20 plants of each genotype and error bars represent ± SD. Statistical significance was determined by Student’s *t*-tests and significant differences (*P* < 0.05 and *P* < 0.01) are indicated by * and ** respectively. Experiments are performed more than three times and result is the representative of one set.

To determine whether cadmium tolerance could be enhanced by overexpressing *SaREFl in planta*, we transformed wild-type *Arabidopsis* with a construct in which *SaREFl* was driven by the 35S promoter. Seven independent T3 homozygous transgenic lines were obtained (OE-1 to OE-7) and the expression of *SaREFl* in different transgenic plants was assessed using RT-PCR. The results showed that the expression of *S. alfredii SaREFl* gene could be detected in all lines (**Figure 5A**), and three independent transgenic lines (OE-1, OE-2, and OE-3) were chosen for further tolerance study. Seeds of WT and *SaREFl* expressing *Arabidopsis* lines were germinated and grown on ½ MS media with or without cadmium. Seedlings were sampled for the measurement of root length and FW, which were considered as tolerance/sensitivity parameters for WT and transgenic lines. Under normal growing conditions, no significant difference was observed between the WT and the *SaREFl* expressing seedlings (**Figure 5B**). When grown on ½ MS media containing 100 μM CdCl_2_, both WT and *SaREFl* expressing seedlings exhibited similar reduced growth. However, transgenic lines showed greater tolerance to Cd stress (**Figure 5B**). Quantitative analysis showed that root length and FW of the *SaREFl*-expressing seedlings were significantly (*P* < 0.05) higher than those of WT (**Figure 5C**). Moreover, the weight of whole seedlings was also measured and more biomass was observed in the transgenic lines in comparison to WT plants grown on medium containing 100 μM CdCl_2_ (**Figure 5D**). The above findings suggest that *SaREFl*-expressing seedlings were more tolerant to cadmium stress comparing to wild type plants.

**FIGURE 6:**
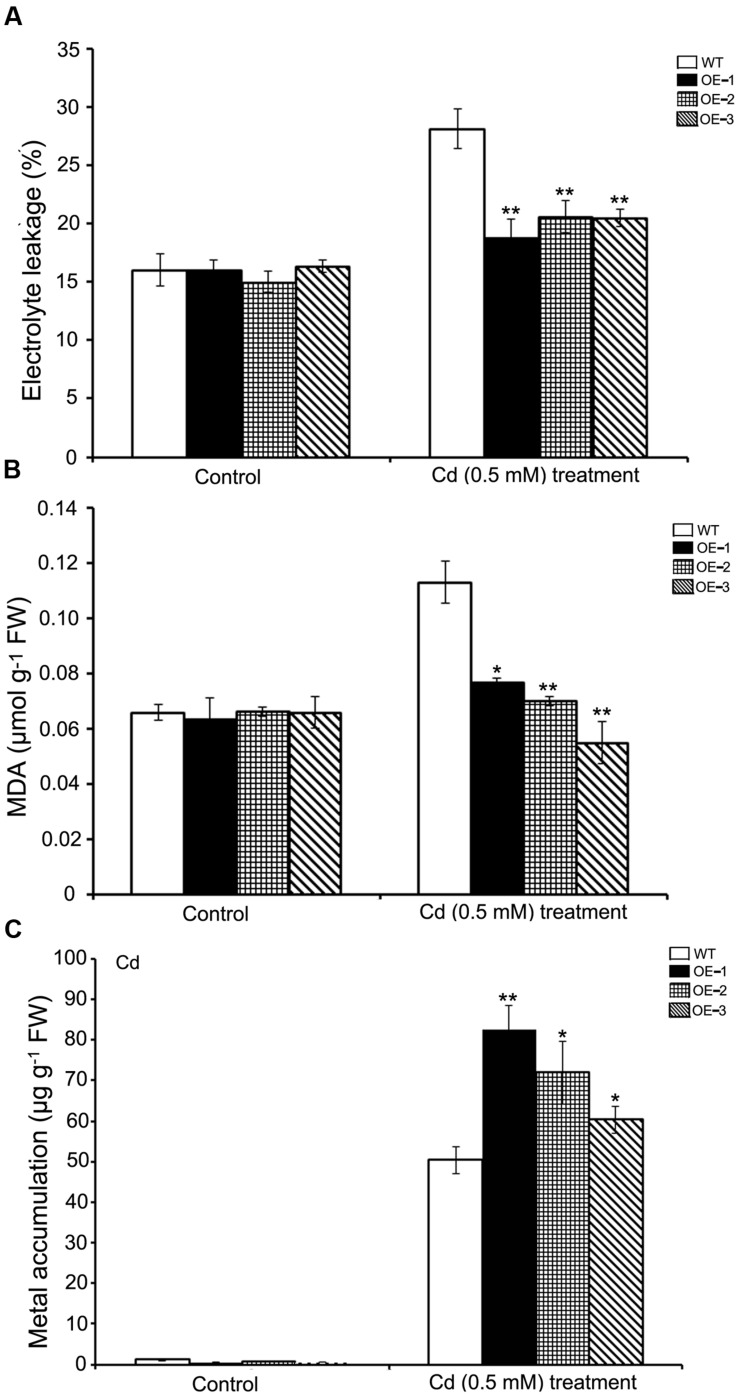
**Analysis of *SaREFl* transgenic *Arabidopsis* lines under cadmium stress condition. (A)** Measurement of electrolyte leakage of WT and transgenic lines (OE-1, OE-2, and OE-3); **(B)** Estimation of MDA in WT and transgenic lines (OE-1, OE-2, and OE-3); **(C)** Assessment of Cd accumulation in WT and transgenic lines (OE-1, OE-2, and OE-3) under stress conditions. Values are the average of three independent experiments error bars represent _SD (*P, 0.05; **P, 0.01).

For the purpose of verifying whether *SaREFl* could confer cadmium tolerance under natural conditions, we grew WT and *SaREFl*-expressing *Arabidopsis* lines on soil for 3 weeks and then irrigated the plants with 500 μM CdCl_2_ for an additional 10 days. Two physiological indexes of plant indicating cell membrane stability and reactive oxygen species damage were assessed to monitor the growing conditions. The EL of both transgenic lines and WT showed an increase caused by the stress treatment. However, transgenic lines overexpressing *SaREFl* displayed significantly reduced EL values compared to WT plants (**Figure 6A**) implying the possible role of *SaREFl* gene in cell membrane protection. It was further supported by lipid peroxidation analysis, in which high MDA contents were found in WT plants compared to *SaREFl*-expressing transgenic lines under stress condition (**Figure 6B**). Furthermore, quantification of Cd contents in the aboveground plant parts was performed to study whether tolerance is accompanied with accumulation of heavy metals. The results showed that the three *SaREFl*-expressing *Arabidopsis* lines accumulated more Cd compared to WT (**Figure 6C**). Taken these results together, these observations supported that expression of *SaREFl* in *Arabidopsis* is related to Cd tolerance.

### Characterization of SaREFl-Interacting Proteins

**FIGURE 7:**
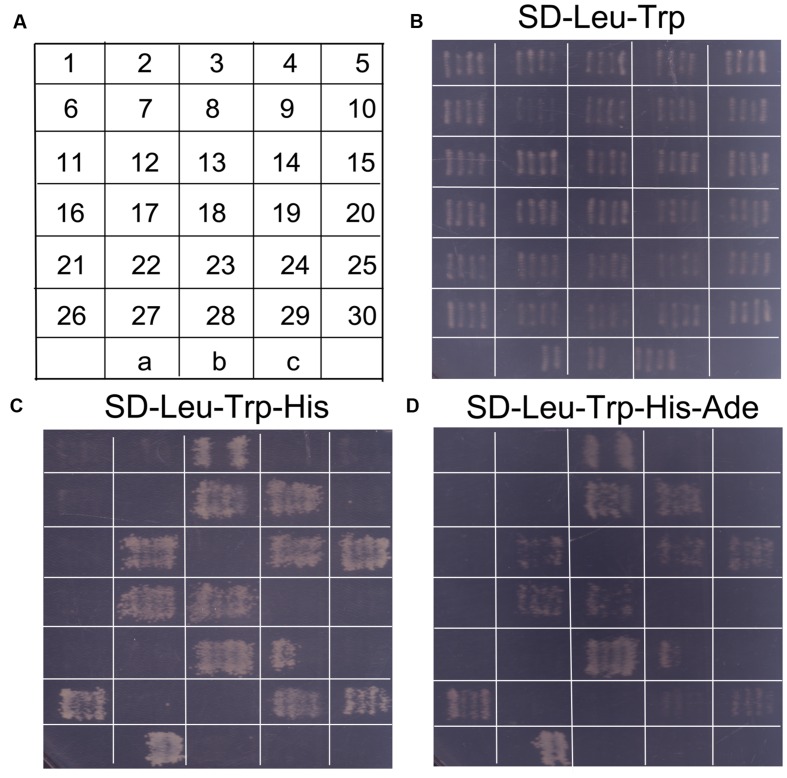
**Screening of probable proteins interacting with *SaREFl* was performed through yeast two-hybrid technique. (A)** The diagram displayed continuous numbering representing for different colonies screened by yeast mating. “a” represented for positive control (pGBKT7-53 interacting with pGADT7-T); “b” represented for negative control (pGBKT7-Lam interacting with pGADT7-T); “c” represented for self-activation control (pGBKT7-REF interacting with pGADT7). **(B)** Screened colonies grew on medium SD-Leu-Trp; **(C)** Screened colonies grew on medium SD-Leu-Trp-His; **(D)** Screened colonies grew on medium SD-Leu-Trp-His-Ade.

In order to identify potential proteins interacting with SaREFl, we screened a *S. alfredii* Hance (HE) cDNA library using yeast two-hybrid technology with SaREFl as bait. Based on the results of self-activation assay, *SaREFl* did not self-activate the expression of *His* and *Ade* reporter genes. An initial screen was performed under stringent selective conditions for HIS3 activity and those colonies that could grow on SD-Leu-Trp-His medium were further tested for the activation of the *Ade* reporter gene. The screening among cDNA library produced prey inserts of 30 strongly growing colonies (**Figure 7B**). These His^+^ and Ade^+^ positive colonies were sequenced, among which nine colonies contained sequences encoding the prenylated Rab acceptor (*PRA1*) family proteins.

To determine the positive interactions, the bait (pGBKT7 + *SaREFl*) and the rescued prey plasmids were re-transformed into yeast Y2HGold. Subsequently, 12 independent colonies that could activate both the *His* reporter gene and the *Ade* reporter genes were distinguished from false positive interactions (**Figures 7C,D**). Among them, eight colonies were sequenced to be the prenylated Rab acceptor (*PRA1*) family proteins numbered as 3, 8, 9, 12, 14, 15, 17, 18, respectively as depicted in **Figure 7A**. The rest were COP9 signalosome complex subunit 5 (XP_008445090.1, numbered as 23), *REF/SRPP*-like protein (XP_008389863.1, numbered as 24), NAD(P)-binding Rossmann-fold superfamily protein isoform 1 (XP_007040707.1, numbered as 26), chromo domain protein (XP_009776768.1, numbered as 29) and binding partner of ACD11 1 (XP_004491782.1, numbered as 30).

### Subcellular Localization of SaREFl

**FIGURE 8:**
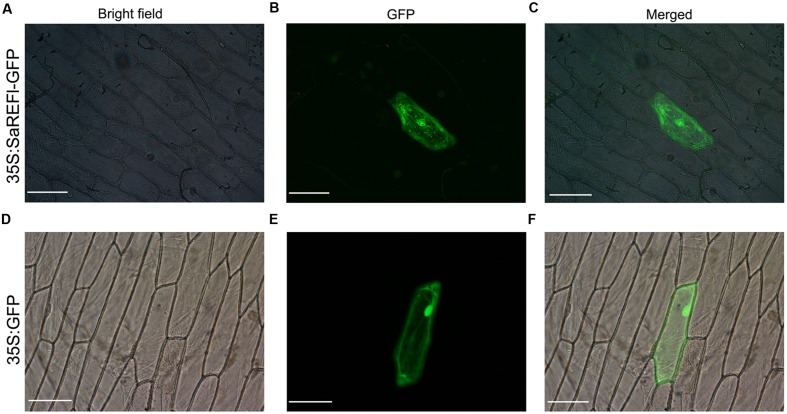
**Subcellular localization of *SaREFl* in onion epidermal cells. (A–C)** *SaREFl* fused to GFP was transiently expressed in onion epidermal cells. **(D–F)** GFP alone was transiently expressed in epidermal cells. **(A,D)** Bright filed images; **(B,E)** GFP fluorescence images; **(C,F)** Artificially merged images. Bars, 100 μm.

The localization of *SaREFl* protein was predicted using the TargetP program^1^ which inferred that it was mainly orientated in the cytosol. We fused the coding region of *SaREFl* at the N-terminus of a green fluorescent protein (GFP) fragment under the control of the CaMV 35S promoter. Subcellular localization of SaREF1-GFP fusion protein was examined by the biolistic assay in onion epidermal cell. Visualized fluorescence indicated that the fluorescent signals of SaREFl-GFP signal were dispersed throughout the cell similar to free GFP (**Figures 8D–F**), however, some patchy spots in cytosol were detected suggesting potential association with organelles for its cellular functions (**Figures 8A–C**).

## Discussion

Heavy-metal environmental pollution is gradually becoming a serious and threating issue confronting society, scientists, and regulators worldwide (Nwaichi et al., 2014). While humans and animals still have an opportunity to avoid heavy metal-contaminated areas as they can move freely, plants cannot. Hence they need to evolve ways to combat the heavy metals they encounter in their living environment. Various defense mechanisms have been developed to prevent heavy-metal-induced injuries, including controlling influx and eﬄux of metals, binding metals to the cell wall, prohibiting toxic metal accumulation in sensitive tissues, chelation by organic molecules and sequestration in vacuoles (Verbruggen et al., 2009b; Tang et al., 2010; Álvarez et al., 2012). These mechanisms, which help plants to sustain their cellular homeostasis, are the outcome of long-term evolution and natural selection. Evolving to adaption is a path of survival living beings adopted to earn a place in the nature. The hyper-accumulating ecotype of *Sedum alfredii* Hance was not an exception either. As an organism inhabiting in the deserted Pb/Zn mine district, the traits of heavy-metal hyper-accumulating and resistance were precious gifts endowed by nature. Although no phylogenetic analysis was performed to explore gene evolution in this species, it is hypothesized that there would be numerous genes evolving and obtaining novel functions to cope with the severe environment.

Rubber elongation factor initially received most attention as the major membrane protein of rubber particles and its intervention in the coagulation of rubber particles was regarded as via mechanisms of agglutination or aggregation more than via an enzymatic activity (Berthelot et al., 2012). But there is also no convincing evidence to exclude the possibility of REF participating in the recruitment of rubber enzymes to the surface of latex globules (Berthelot et al., 2012). *REF* was found to be associated with latex allergy with several *IgE* epitopes, although its role in the immune response leading to latex hypersensitivity has not yet been determined (Banerjee et al., 2000). Although *REF* had been found to exhibit significant matches with *SRP*, the direct role of *REF* in combating stress has rarely been reported.

In our previous study, a transcript showing high homology to *REF* was detected to enhance yeast tolerance to cadmium. Preliminary bioinformatics analysis including phylogenetic clustering indicated that it was more identical to *REFs* in other species rather than *SRPs*. Therefore, we designated it as *SaREFl* and focused on the elucidation of the role it might play in cadmium tolerance in this study.

There have been numerous reports demonstrating that expression of genes related to stress could be induced by stress treatment such as transporters, heat-shock proteins, metallothioneins and sulfate-metabolizing proteins (Zhang et al., 2001; Norton et al., 2008; Song et al., 2010; Kumar et al., 2013, 2015; Li et al., 2014). In our study, *SaREFl* expression profiles were investigated in *S. alfredii* plants exposed to different heavy metals treatments. It was found that *SaREFl* exhibited different modes of transcriptional regulation upon heavy metal excess and the most sensitive tissue was the root. The transcript abundance of *SaREFl* demonstrated strong elevation in response to Cd, moderate up-regulation to Pb and Cu, but no significant change in response to Zn. Based on the above results, it could be observed that although *SaREFl* was not endowed with a tissue-specific expression pattern (data not shown), however, it did exist a tissue-priority or preference in the response to the stress. This may be partly due to the facts that roots are the primary site of perception and injury when the plants were confronted with stresses. Moreover, *SaREFl* exhibited a differential regulation to different heavy metal irons. It was somewhat surprising that it showed no dramatic response to Zn stress although *S. alfredii* Hance (HE) is a hyperaccumulator of Zn and Cd. These findings provide us potential research directions, more significantly, to a large degree, it may be an exhibition of heavy-metal resistance characteristics of *SaREFl*.

To investigate the cellular function of *SaREFl*, the gene was expressed in *S. cerevisiae* strain DTY167 (*ycf1*), a cadmium-sensitive yeast stain resulted from the deletion of the yeast cadmium factor gene (*YCF1*) (Szczypka et al., 1994; Li et al., 1996). Using this Cd-sensitive strain (*ycf1*), it was found that the growth of Δ*ycf1* mutant transformed with the empty vector was inhibited by Cd whereas cells harboring the *SaREFl* expression vector exhibited better vitality. The results suggested that *SaREFl* was able to complement Cd sensitivity and rescued or partially rescued the Cd -sensitivity phenotype in the mutant yeast strain, which further supported the role of *SaREFl* in Cd tolerance. Moreover, metal tolerance assays using the yeast strain INVSc1 showed that expression of the *SaREFl* gene in yeast cells exhibiting a certain specificity to iron provided no obvious tolerance to Cu^2+^, Pb^2+^ or Zn^2+^. Although it was hard to assess the precise function of *SaREFl* into specific categories such as uptake or translocation, we inferred that the *SaREFl*-induced Cd tolerance may be due to the exertion of direct effects to several major pathways or taking parts in assembling other targets that contribute to cadmium tolerance. To address this question, the analysis of differential gene expression patterns present in *SaREFl*-expressing and wild-type yeast at the whole genome level needed to be investigated, which would also enable the characterization of novel targets of *SaREFl* and may uncover new aspects of Cd tolerance.

The trait of enhancing cadmium tolerance was further investigated through overexpression of *SaREFl* in *Arabidopsis*. Whereas retarded growth occurred in both the wild type and the transgenic lines in Cd-containing media, quantification of the FW and root length of seedlings confirmed that a significant difference could be observed between them, suggesting that the transgenic lines displayed a higher level of tolerance than wild types. Under stress, mature transgenic plant lines overexpressing *SaREFl* performed much better than wild types as far as the measurements of cell membrane stability indicated by electrolyte leakage and lipid peroxidation (MDA content). Moreover, in the transgenic lines, significantly higher accumulation of Cd was found in comparison to wild type. It is well known that the metabolic homestasis of a cell would be disturbed by abiotic stress resulting in a cascade of events including the enhanced production of ROS and the damage of cell membranes (Verbruggen et al., 2009b; Zwiewka et al., 2015). The findings here seemed to support a role of *SaREFl* in cadmium tolerance which is possibly achieved through maintaining the metabolic homeostasis during exposure to heavy metal stress.

Although the function of *SaREFl* in Cd tolerance has been assessed by heterologous expression, how *SaREFl* acts in cadmium tolerance is still unclear. The confocal microscopic analysis of the *SaREFl-*GFP protein confirmed that *SaREFl* was localized to the cytosol, suggesting that it might play roles in intercellular processes and alleviate the cytological effects of CdCl_2_ stress. An important intracellular metal detoxification is accomplished through metal chelation by peptide ligands such as metallothioneins (MT) and phytochelatins (PCs) (Rascio and Navari-Izzo, 2011). Metallothioneins (MTs) coordinate heavy-metal ions owing to the metal-thiolate bonds contributed by their abundant cysteine residues (Gil-Moreno et al., 2015) while phytochelatins (PCs) are enzymatically synthesized peptides consisting of only three amino acids, glutamine (Glu), cysteine (Cys), and glycine (Gly) with the Glu, and Cys residues linked through a γ-carboxylamide bond (Pal and Rai, 2010). The aminoacid sequence of *SaREFl* contained only two cysteine residues and lacked metal binding motifs. Hence, it would be hard to classify *SaREFl* as chelators. In order to characterize possible target genes interacting with *SaREFl*, we performed a screening among *S. alfredii* cDNA library using the yeast two-hybrid technology with SaREFl as a bait. We found that a gene named *Prenylated Rab acceptor 1 (PRA1) domain protein* was detected with high frequency. Numerous studies have shown that prenylated rab acceptor 1 *(PRA1)* domain proteins may functionally link various vesicle trafficking proteins, regulate vesicle trafficking and participate in protein transport through the Golgi apparatus (Bucci et al., 1999; Lin et al., 2001; Sivars et al., 2003; Kamei et al., 2008; Heo et al., 2010, Jung et al., 2011). Vesicular trafficking is an important step involved in the secretory process (Kohorn, 2000, Willats et al., 2001; Lee et al., 2008; Vassilieva and Nusrat, 2008). This dynamic homeostasis could be influenced by a variety of stimuli, for instance, various stress factors (Örvar et al., 2000; Halperin et al., 2003; Fan et al., 2011). The interaction of SaREFl with PRA suggested that *SaREFl* might possibly get involved in regulation or recovery of vesicle trafficking to combat the imposed stress. Although it still needs further validation by other protein-interaction techniques, it does provide some insight into the precise mechanism of *SaREFl*-related to Cd tolerance.

In summary, the present study assessed the characteristics of *SaREFl* associated with Cd tolerance. Several findings of interest include the different modes of transcriptional regulation of *SaREFl* upon heavy metal excess. Although there are still many puzzles around *SaREFl*, it seems that *SaREFl* is endowed with novel functions alongside the evolution of *S. alfredii* Hance (HE) being a Cd/Zn co-hyperaccumulator. The findings here could contribute toward enriching the connotation of *REF-like* genes and provide theoretical guidance for the application in breeding heavy metal-tolerant crop plants.

## Author Contributions

RZ and MyL planned and designed the research. MyL, WQ, XS and XH performed experiments. LZ, XH, JJ, GQ, JS and MqL contributed analytical tools. MyL wrote the paper. All authors read and approved the manuscript.

## Conflict of Interest Statement

The authors declare that the research was conducted in the absence of any commercial or financial relationships that could be construed as a potential conflict of interest.

## Footnotes

1 http://www.cbs.dtu.dk/services/TargetP

## Supplementary Material

FIGURE S1 **Assessment of metal tolerance of *SaREFl* to Zn^2+^, Cu^2+^ and Pb^2+^**. Two lines of yeast cells expressing *SaREFl* or empty vector were cultured on medium supplemented with 5 mM Cu^2+^, 1.5 mM Pb^2+^ and 15 mM Zn^2+^.
