# Supplementary figures and images for: Functional Characterization of a Gene in Sedum alfredii Hance Resembling Rubber Elongation Factor Endowed with Functions Associated with Cadmium Tolerance
Source: Front Plant Sci. 2016 Jun 29;7:965. doi: 10.3389/fpls.2016.00965 (PMC4925709; doi:10.3389/fpls.2016.00965)

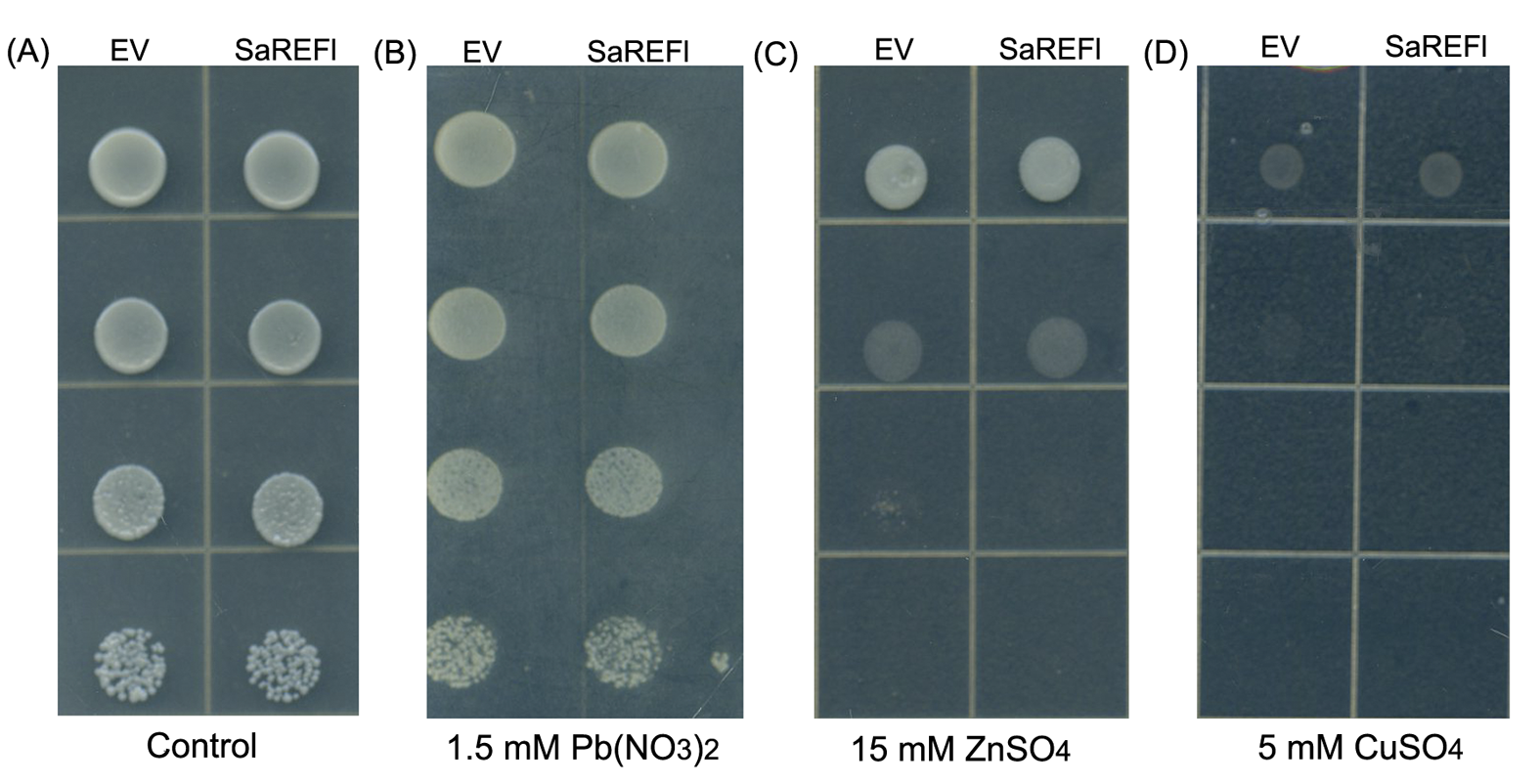

Supplement: FIGURE S1 — Assessment of metal tolerance of SaREFl to Zn2+, Cu2+ and Pb2+. Two lines of yeast cells expressing SaREFl or empty vector were cultured on medium supplemented with 5 mM Cu2+, 1.5 mM Pb2+ and 15 mM Zn2+. [file Image_1.TIF]
